# (4-Chloro­benzoato)bis(5-methyl-2-pyridylamine)silver(I)

**DOI:** 10.1107/S1600536808008064

**Published:** 2008-03-29

**Authors:** Yong-Qiang Tian, Tong Shen, Wu-Xia Liu

**Affiliations:** a School of Chemical and Biological Engineering, Lanzhou Jiaotong University, Lanzhou 730070, People’s Republic of China

## Abstract

The title compound, [Ag(C_7_H_4_ClO_2_)(C_6_H_8_N_2_)_2_], is a mononuclear silver(I) complex. The Ag^I^ atom is three-coordinated by two pyridine N atoms from two 5-methyl­pyridin-2-ylamine ligands and by one O atom of a 4-chloro­benzoate ligand, forming a distorted T-shaped coordination. In the crystal structure, the mol­ecules are linked through inter­molecular N—H⋯O hydrogen bonds, forming chains running along the *b* axis.

## Related literature

For related literature, see: Bi *et al.* (2002 ▶); Deng *et al.* (2004 ▶); Jones *et al.* (2006 ▶); Khan *et al.* (2005 ▶); Kristiansson (2000 ▶); Li *et al.* (2007 ▶); Odoko *et al.* (2007 ▶); Sailaja *et al.* (2001 ▶); Wang & Okabe (2004 ▶).
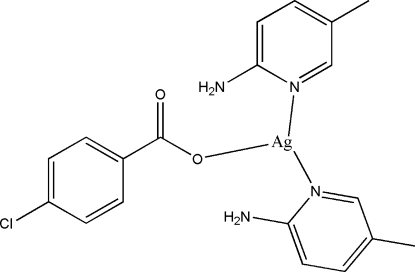


## Experimental

### 

#### Crystal data

[Ag(C_7_H_4_ClO_2_)(C_6_H_8_N_2_)_2_]
*M* *_r_* = 479.71
Monoclinic, 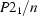

*a* = 15.983 (3) Å
*b* = 5.7428 (9) Å
*c* = 21.703 (4) Å
β = 98.460 (2)°
*V* = 1970.4 (6) Å^3^
*Z* = 4
Mo *K*α radiation
μ = 1.18 mm^−1^
*T* = 298 (2) K
0.37 × 0.35 × 0.32 mm

#### Data collection

Bruker SMART CCD area-detector diffractometer
Absorption correction: multi-scan (*SADABS*; Sheldrick, 1996 ▶) *T*_min_ = 0.669, *T*_max_ = 0.704
13465 measured reflections
4064 independent reflections
3277 reflections with *I* > 2σ(*I*)
*R*_int_ = 0.032

#### Refinement

*R*[*F* ^2^ > 2σ(*F* ^2^)] = 0.036
*wR*(*F* ^2^) = 0.087
*S* = 1.03
4064 reflections
258 parameters
6 restraints
H atoms treated by a mixture of independent and constrained refinement
Δρ_max_ = 0.53 e Å^−3^
Δρ_min_ = −0.34 e Å^−3^

### 

Data collection: *SMART* (Bruker, 1998 ▶); cell refinement: *SAINT* (Bruker, 1998 ▶); data reduction: *SAINT*; program(s) used to solve structure: *SHELXS97* (Sheldrick, 2008 ▶); program(s) used to refine structure: *SHELXL97* (Sheldrick, 2008 ▶); molecular graphics: *SHELXTL* (Sheldrick, 2008 ▶); software used to prepare material for publication: *SHELXTL*.

## Supplementary Material

Crystal structure: contains datablocks global, I. DOI: 10.1107/S1600536808008064/ci2572sup1.cif

Structure factors: contains datablocks I. DOI: 10.1107/S1600536808008064/ci2572Isup2.hkl

Additional supplementary materials:  crystallographic information; 3D view; checkCIF report

## Figures and Tables

**Table:** 

Ag1—N1	2.179 (2)
Ag1—N3	2.193 (2)
Ag1—O1	2.647 (2)

**Table:** 

N1—Ag1—N3	151.99 (9)
N1—Ag1—O1	103.05 (9)
N3—Ag1—O1	104.89 (9)

**Table 2:** Hydrogen-bond geometry (Å, °)

*D*—H⋯*A*	*D*—H	H⋯*A*	*D*⋯*A*	*D*—H⋯*A*
N2—H2*A*⋯O1	0.88 (3)	2.11 (3)	2.977 (4)	169 (4)
N2—H2*B*⋯O2^i^	0.89 (1)	1.94 (1)	2.822 (4)	174 (3)
N4—H4*A*⋯O1	0.88 (3)	2.09 (3)	2.966 (4)	167 (4)
N4—H4*B*⋯O1^ii^	0.88 (1)	2.09 (3)	2.955 (3)	167 (3)

Symmetry codes: (i) 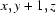; (ii) 
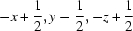
.

**Fig. 1.**
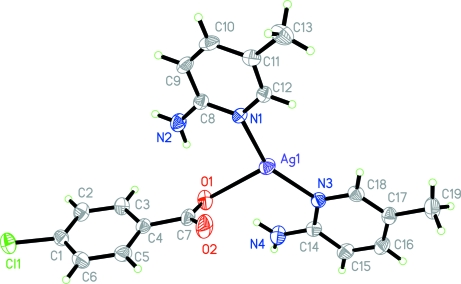
The structure of the title complex, showing 30% probability displacement ellipsoids and the atomic numbering.

**Fig. 2.**
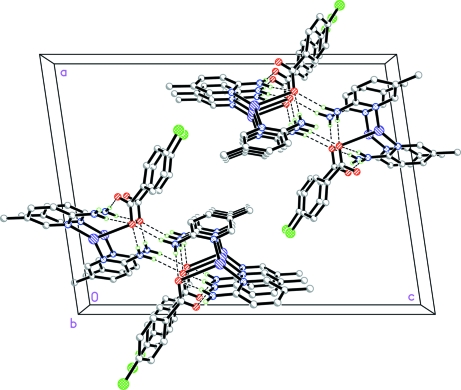
The crystal packing of the title compound, viewed down the b axis. Intermolecular hydrogen bonds are shown as dashed lines.

## supplementary crystallographic information

### Comment

Silver(I) complexes with carboxylate ligands and amine compounds have been widely investigated due to their versatile structures (Odoko *et al.*, 2007; Li *et al.*, 2007; Jones *et al.*, 2006; Bi *et al.*, 2002). We report herein the crystal structure of the title silver(I) complex.

The title compound is a mononuclear silver(I) complex (Fig. 1). The Ag^I^ atom is three-coordinated by two pyridine N atoms from two 5-methylpyridin-2-ylamine ligands and by one O atom of a 4-chlorobenzoate ligand, forming a distorted T-shaped coordination, the distortion being caused by the weak coordination of the carboxylate O atom (Ag1—O1 = 2.647 (2) Å, Table 1). The Ag—N bond lengths (Table 1) are comparable with the values observed in other silver(I) complexes (Kristiansson, 2000; Wang & Okabe, 2004; Sailaja *et al.*, 2001; Khan *et al.*, 2005; Deng *et al.*, 2004).

In the crystal structure, the molecules are linked through intermolecular N—H···O hydrogen bonds (Table 2), forming chains running along the *b* axis (Fig. 2).

### Experimental

Ag_2_O (0.1 mmol, 23.2 mg) and 4-chlorobenzoic acid (0.1 mmol, 15.6 mg) were dissolved in an ammonia solution (10 ml, 30%), and the mixture was stirred for 20 min at room temperature. To the above mixture was added with stirring a methanol solution (3 ml) of 5-methylpyridin-2-ylamine (0.2 mmol, 21.6 mg). The final mixture was stirred for 30 min at room temperature. The resulting clear colourless solution was kept in dark for 12 d, yielding colourless block-shaped crystals.

### Refinement

Atoms H2A, H2B, H4A and H4B were located in a difference Fourier map and refined isotropically, with N—H and H···H distances restrained to 0.90 (1) Å and 1.43 (2) Å, respectively. Other H atoms were placed in idealized positions and constrained to ride on their parent atoms, with C—H distances in the range 0.93–0.96 Å, and with *U*_iso_(H) = 1.2 or 1.5*U*_eq_(C).

### Crystal data

**Table:**

[Ag(C_7_H_4_ClO_2_)(C_6_H_8_N_2_)_2_]	*F*_000_ = 968
*M**_r_* = 479.71	*D*_x_ = 1.617 Mg m^−^^3^
Monoclinic, *P*2_1_/*n*	Mo *K*α radiation λ = 0.71073 Å
Hall symbol: -P 2yn	Cell parameters from 3734 reflections
*a* = 15.983 (3) Å	θ = 2.5–24.9º
*b* = 5.7428 (9) Å	µ = 1.18 mm^−^^1^
*c* = 21.703 (4) Å	*T* = 298 (2) K
β = 98.460 (2)º	Block, colourless
*V* = 1970.4 (6) Å^3^	0.37 × 0.35 × 0.32 mm
*Z* = 4	

### Data collection

**Table:**

Bruker SMART CCD area-detector diffractometer	4064 independent reflections
Radiation source: fine-focus sealed tube	3277 reflections with *I* > 2σ(*I*)
Monochromator: graphite	*R*_int_ = 0.032
*T* = 298(2) K	θ_max_ = 26.5º
ω scans	θ_min_ = 1.5º
Absorption correction: multi-scan(SADABS; Sheldrick, 1996)	*h* = −19→20
*T*_min_ = 0.669, *T*_max_ = 0.704	*k* = −7→7
13465 measured reflections	*l* = −26→27

### Refinement

**Table:**

Refinement on *F*^2^	Secondary atom site location: difference Fourier map
Least-squares matrix: full	Hydrogen site location: inferred from neighbouring sites
*R*[*F*^2^ > 2σ(*F*^2^)] = 0.036	H atoms treated by a mixture of independent and constrained refinement
*wR*(*F*^2^) = 0.087	*w* = 1/[σ^2^(*F*_o_^2^) + (0.0422*P*)^2^ + 0.4221*P*] where *P* = (*F*_o_^2^ + 2*F*_c_^2^)/3
*S* = 1.03	(Δ/σ)_max_ = 0.001
4064 reflections	Δρ_max_ = 0.53 e Å^−^^3^
258 parameters	Δρ_min_ = −0.34 e Å^−^^3^
6 restraints	Extinction correction: none
Primary atom site location: structure-invariant direct methods	

### Special details

**Table:**

Geometry. All e.s.d.'s (except the e.s.d. in the dihedral angle between two l.s. planes) are estimated using the full covariance matrix. The cell e.s.d.'s are taken into account individually in the estimation of e.s.d.'s in distances, angles and torsion angles; correlations between e.s.d.'s in cell parameters are only used when they are defined by crystal symmetry. An approximate (isotropic) treatment of cell e.s.d.'s is used for estimating e.s.d.'s involving l.s. planes.
Refinement. Refinement of *F*^2^ against ALL reflections. The weighted *R*-factor *wR* and goodness of fit *S* are based on *F*^2^, conventional *R*-factors *R* are based on *F*, with *F* set to zero for negative *F*^2^. The threshold expression of *F*^2^ > σ(*F*^2^) is used only for calculating *R*-factors(gt) *etc*. and is not relevant to the choice of reflections for refinement. *R*-factors based on *F*^2^ are statistically about twice as large as those based on *F*, and *R*- factors based on ALL data will be even larger.

### Fractional atomic coordinates and isotropic or equivalent isotropic displacement parameters (Å^2^)

**Table:**

	*x*	*y*	*z*	*U*_iso_*/*U*_eq_	
Ag1	0.291478 (15)	0.09298 (4)	0.077611 (11)	0.05012 (11)	
Cl1	0.69700 (7)	0.65902 (19)	0.36548 (5)	0.0793 (3)	
O1	0.35007 (14)	0.2179 (4)	0.19330 (10)	0.0573 (6)	
O2	0.45197 (17)	0.0110 (5)	0.16007 (13)	0.0760 (8)	
N1	0.34955 (14)	0.3452 (4)	0.02193 (11)	0.0388 (5)	
N2	0.39194 (19)	0.5847 (5)	0.10583 (14)	0.0556 (7)	
N3	0.20646 (14)	−0.2005 (4)	0.08709 (11)	0.0432 (6)	
N4	0.2241 (2)	−0.1612 (6)	0.19393 (13)	0.0605 (8)	
C1	0.6161 (2)	0.5050 (6)	0.31981 (14)	0.0507 (8)	
C2	0.5373 (2)	0.6012 (6)	0.30750 (15)	0.0542 (8)	
H2	0.5258	0.7432	0.3251	0.065*	
C3	0.4749 (2)	0.4848 (6)	0.26869 (14)	0.0491 (7)	
H3	0.4210	0.5489	0.2602	0.059*	
C4	0.49170 (19)	0.2737 (5)	0.24224 (13)	0.0427 (7)	
C5	0.5713 (2)	0.1802 (6)	0.25682 (16)	0.0554 (8)	
H5	0.5831	0.0371	0.2400	0.066*	
C6	0.6338 (2)	0.2935 (7)	0.29568 (17)	0.0631 (9)	
H6	0.6873	0.2277	0.3054	0.076*	
C7	0.4262 (2)	0.1546 (6)	0.19559 (14)	0.0481 (8)	
C8	0.38570 (17)	0.5444 (5)	0.04449 (15)	0.0417 (7)	
C9	0.41727 (18)	0.7050 (5)	0.00414 (17)	0.0501 (8)	
H9	0.4412	0.8445	0.0198	0.060*	
C10	0.41269 (19)	0.6557 (6)	−0.05702 (17)	0.0525 (8)	
H10	0.4337	0.7613	−0.0834	0.063*	
C11	0.37643 (19)	0.4460 (6)	−0.08121 (15)	0.0465 (7)	
C12	0.34602 (17)	0.3021 (5)	−0.03981 (13)	0.0414 (7)	
H12	0.3209	0.1636	−0.0551	0.050*	
C13	0.3731 (2)	0.3791 (7)	−0.14840 (16)	0.0674 (11)	
H13A	0.3471	0.2288	−0.1553	0.101*	
H13B	0.4295	0.3734	−0.1586	0.101*	
H13C	0.3406	0.4923	−0.1743	0.101*	
C14	0.19164 (17)	−0.2802 (6)	0.14244 (14)	0.0438 (7)	
C15	0.1459 (2)	−0.4866 (6)	0.14600 (16)	0.0535 (8)	
H15	0.1354	−0.5405	0.1845	0.064*	
C16	0.1171 (2)	−0.6072 (6)	0.09412 (17)	0.0548 (8)	
H16	0.0872	−0.7447	0.0970	0.066*	
C17	0.13206 (19)	−0.5268 (6)	0.03557 (15)	0.0493 (8)	
C18	0.17643 (19)	−0.3242 (6)	0.03562 (15)	0.0477 (7)	
H18	0.1868	−0.2669	−0.0026	0.057*	
C19	0.1021 (3)	−0.6605 (7)	−0.02362 (18)	0.0726 (11)	
H19A	0.1096	−0.5667	−0.0590	0.109*	
H19B	0.0433	−0.6984	−0.0253	0.109*	
H19C	0.1344	−0.8013	−0.0241	0.109*	
H4B	0.209 (2)	−0.185 (6)	0.2308 (9)	0.080*	
H4A	0.255 (2)	−0.032 (4)	0.1940 (16)	0.080*	
H2B	0.407 (2)	0.720 (3)	0.1241 (14)	0.080*	
H2A	0.374 (2)	0.489 (5)	0.1330 (13)	0.080*	

### Atomic displacement parameters (Å^2^)

**Table:**

	*U*^11^	*U*^22^	*U*^33^	*U*^12^	*U*^13^	*U*^23^
Ag1	0.05372 (17)	0.04898 (17)	0.04972 (16)	−0.01056 (11)	0.01445 (11)	0.00527 (11)
Cl1	0.0798 (7)	0.0821 (7)	0.0669 (6)	−0.0191 (5)	−0.0192 (5)	−0.0079 (5)
O1	0.0522 (13)	0.0746 (17)	0.0462 (13)	−0.0174 (12)	0.0110 (10)	−0.0097 (12)
O2	0.0837 (18)	0.0637 (16)	0.0788 (18)	−0.0047 (14)	0.0062 (14)	−0.0372 (15)
N1	0.0404 (13)	0.0345 (13)	0.0426 (14)	−0.0033 (10)	0.0100 (10)	0.0007 (11)
N2	0.0648 (18)	0.0463 (17)	0.0552 (18)	−0.0051 (14)	0.0075 (14)	−0.0123 (14)
N3	0.0432 (13)	0.0454 (15)	0.0419 (14)	−0.0063 (11)	0.0090 (11)	0.0021 (12)
N4	0.0729 (19)	0.070 (2)	0.0420 (16)	−0.0249 (16)	0.0199 (14)	−0.0044 (15)
C1	0.060 (2)	0.054 (2)	0.0359 (16)	−0.0103 (17)	−0.0015 (14)	0.0007 (15)
C2	0.066 (2)	0.052 (2)	0.0465 (18)	−0.0065 (17)	0.0145 (16)	−0.0162 (15)
C3	0.0491 (18)	0.0522 (19)	0.0472 (18)	−0.0026 (15)	0.0113 (14)	−0.0094 (15)
C4	0.0540 (17)	0.0401 (17)	0.0349 (15)	−0.0079 (14)	0.0098 (13)	0.0005 (13)
C5	0.070 (2)	0.0414 (18)	0.053 (2)	0.0066 (17)	0.0023 (16)	−0.0042 (16)
C6	0.062 (2)	0.057 (2)	0.065 (2)	0.0071 (18)	−0.0105 (17)	0.0010 (19)
C7	0.063 (2)	0.0435 (18)	0.0385 (17)	−0.0144 (15)	0.0093 (15)	−0.0058 (14)
C8	0.0382 (15)	0.0341 (17)	0.0530 (18)	0.0034 (12)	0.0073 (13)	−0.0022 (14)
C9	0.0438 (17)	0.0309 (17)	0.076 (2)	0.0001 (13)	0.0093 (15)	0.0040 (16)
C10	0.0420 (17)	0.049 (2)	0.069 (2)	0.0037 (14)	0.0166 (15)	0.0233 (17)
C11	0.0390 (16)	0.054 (2)	0.0481 (18)	0.0057 (14)	0.0115 (13)	0.0076 (15)
C12	0.0390 (15)	0.0392 (17)	0.0466 (17)	−0.0012 (13)	0.0079 (12)	−0.0022 (14)
C13	0.061 (2)	0.097 (3)	0.046 (2)	0.001 (2)	0.0159 (17)	0.0094 (19)
C14	0.0403 (15)	0.0489 (19)	0.0448 (17)	−0.0016 (14)	0.0147 (13)	0.0035 (15)
C15	0.058 (2)	0.0520 (19)	0.054 (2)	−0.0088 (16)	0.0204 (15)	0.0091 (17)
C16	0.0476 (18)	0.047 (2)	0.072 (2)	−0.0092 (15)	0.0146 (16)	−0.0004 (17)
C17	0.0412 (16)	0.0497 (19)	0.057 (2)	−0.0005 (14)	0.0065 (14)	−0.0085 (16)
C18	0.0474 (17)	0.055 (2)	0.0409 (17)	−0.0020 (15)	0.0085 (13)	0.0045 (15)
C19	0.072 (2)	0.071 (3)	0.071 (3)	−0.008 (2)	−0.001 (2)	−0.021 (2)

### Geometric parameters (Å, °)

**Table:**

Ag1—N1	2.179 (2)	C5—H5	0.93
Ag1—N3	2.193 (2)	C6—H6	0.93
Ag1—O1	2.647 (2)	C8—C9	1.415 (4)
Cl1—C1	1.748 (3)	C9—C10	1.348 (5)
O1—C7	1.263 (4)	C9—H9	0.93
O2—C7	1.240 (4)	C10—C11	1.404 (5)
N1—C8	1.342 (4)	C10—H10	0.93
N1—C12	1.356 (4)	C11—C12	1.362 (4)
N2—C8	1.340 (4)	C11—C13	1.501 (5)
N2—H2B	0.888 (10)	C12—H12	0.93
N2—H2A	0.88 (3)	C13—H13A	0.96
N3—C14	1.339 (4)	C13—H13B	0.96
N3—C18	1.351 (4)	C13—H13C	0.96
N4—C14	1.347 (4)	C14—C15	1.401 (4)
N4—H4B	0.883 (10)	C15—C16	1.344 (5)
N4—H4A	0.88 (3)	C15—H15	0.93
C1—C2	1.366 (5)	C16—C17	1.405 (5)
C1—C6	1.369 (5)	C16—H16	0.93
C2—C3	1.379 (4)	C17—C18	1.363 (5)
C2—H2	0.93	C17—C19	1.513 (5)
C3—C4	1.384 (4)	C18—H18	0.93
C3—H3	0.93	C19—H19A	0.96
C4—C5	1.375 (4)	C19—H19B	0.96
C4—C7	1.510 (4)	C19—H19C	0.96
C5—C6	1.373 (5)		
			
N1—Ag1—N3	151.99 (9)	C10—C9—C8	120.0 (3)
N1—Ag1—O1	103.05 (9)	C10—C9—H9	120.0
N3—Ag1—O1	104.89 (9)	C8—C9—H9	120.0
C8—N1—C12	118.0 (3)	C9—C10—C11	120.5 (3)
C8—N1—Ag1	124.1 (2)	C9—C10—H10	119.7
C12—N1—Ag1	117.89 (19)	C11—C10—H10	119.7
C8—N2—H2B	125 (2)	C12—C11—C10	116.2 (3)
C8—N2—H2A	125 (2)	C12—C11—C13	121.3 (3)
H2B—N2—H2A	110 (2)	C10—C11—C13	122.4 (3)
C14—N3—C18	118.3 (3)	N1—C12—C11	125.1 (3)
C14—N3—Ag1	122.7 (2)	N1—C12—H12	117.5
C18—N3—Ag1	118.44 (19)	C11—C12—H12	117.5
C14—N4—H4B	123 (2)	C11—C13—H13A	109.5
C14—N4—H4A	125 (2)	C11—C13—H13B	109.5
H4B—N4—H4A	111 (2)	H13A—C13—H13B	109.5
C2—C1—C6	121.4 (3)	C11—C13—H13C	109.5
C2—C1—Cl1	119.4 (3)	H13A—C13—H13C	109.5
C6—C1—Cl1	119.2 (3)	H13B—C13—H13C	109.5
C1—C2—C3	119.1 (3)	N3—C14—N4	118.2 (3)
C1—C2—H2	120.4	N3—C14—C15	120.1 (3)
C3—C2—H2	120.4	N4—C14—C15	121.6 (3)
C2—C3—C4	120.7 (3)	C16—C15—C14	120.5 (3)
C2—C3—H3	119.6	C16—C15—H15	119.8
C4—C3—H3	119.6	C14—C15—H15	119.8
C5—C4—C3	118.4 (3)	C15—C16—C17	120.4 (3)
C5—C4—C7	120.3 (3)	C15—C16—H16	119.8
C3—C4—C7	121.2 (3)	C17—C16—H16	119.8
C6—C5—C4	121.4 (3)	C18—C17—C16	115.9 (3)
C6—C5—H5	119.3	C18—C17—C19	122.3 (3)
C4—C5—H5	119.3	C16—C17—C19	121.8 (3)
C1—C6—C5	118.9 (3)	N3—C18—C17	124.8 (3)
C1—C6—H6	120.6	N3—C18—H18	117.6
C5—C6—H6	120.6	C17—C18—H18	117.6
O2—C7—O1	125.0 (3)	C17—C19—H19A	109.5
O2—C7—C4	117.2 (3)	C17—C19—H19B	109.5
O1—C7—C4	117.7 (3)	H19A—C19—H19B	109.5
N2—C8—N1	118.3 (3)	C17—C19—H19C	109.5
N2—C8—C9	121.4 (3)	H19A—C19—H19C	109.5
N1—C8—C9	120.2 (3)	H19B—C19—H19C	109.5

### Hydrogen-bond geometry (Å, °)

**Table:**

*D*—H···*A*	*D*—H	H···*A*	*D*···*A*	*D*—H···*A*
N2—H2A···O1	0.88 (3)	2.11 (3)	2.977 (4)	169 (4)
N2—H2B···O2^i^	0.89 (1)	1.94 (1)	2.822 (4)	174 (3)
N4—H4A···O1	0.88 (3)	2.09 (3)	2.966 (4)	167 (4)
N4—H4B···O1^ii^	0.88 (1)	2.09 (3)	2.955 (3)	167 (3)

Symmetry codes: (i) *x*, *y*+1, *z*; (ii) −*x*+1/2, *y*−1/2, −*z*+1/2.

## References

[bb1] Bi, W., Sun, D., Cao, R. & Hong, M. (2002). *Acta Cryst.* E**58**, m324–m325.

[bb2] Bruker (1998). *SMART* and *SAINT* Bruker AXS Inc., Madison, Wisconsin, USA.

[bb3] Deng, B., Liu, Z.-D., Liu, X.-Y., Tan, M.-Y. & Zhu, H.-L. (2004). *Acta Cryst.* E**60**, m1444–m1446.

[bb4] Jones, P. G., Crespo, O., Gimeno, M. C. & Laguna, A. (2006). *Acta Cryst.* C**62**, m411–m412. 10.1107/S0108270106028009 16954621

[bb5] Khan, M. A. H., Prasad, T. K. & Rajasekharan, M. V. (2005). *Acta Cryst.* C**61**, m281–m283. 10.1107/S0108270105012448 15930665

[bb6] Kristiansson, O. (2000). *Acta Cryst.* C**56**, 165–167. 10.1107/s0108270199014432 10777874

[bb7] Li, Y.-F., Pan, Z.-H. & Lou, T.-J. (2007). *Acta Cryst.* C**63**, m516–m518. 10.1107/S0108270107048251 17989469

[bb8] Odoko, M., Ise, T. & Okabe, N. (2007). *Acta Cryst.* C**63**, m22–m26. 10.1107/S010827010605102X 17206038

[bb9] Sailaja, S., Swarnabala, G. & Rajasekharan, M. V. (2001). *Acta Cryst.* C**57**, 1162–1165. 10.1107/s0108270101012380 11600772

[bb10] Sheldrick, G. M. (1996). *SADABS* University of Göttingen, Germany.

[bb11] Sheldrick, G. M. (2008). *Acta Cryst.* A**64**, 112–122. 10.1107/S0108767307043930 18156677

[bb12] Wang, Y. & Okabe, N. (2004). *Acta Cryst.* C**60**, m605–m608. 10.1107/S0108270104026502 15579940

